# The Control of the Inebriate

**Published:** 1904-12-17

**Authors:** T. N. Kelynack

**Affiliations:** Honorary Secretary of The British Society for the Study of Inebriety


### HOSPITAL ADMINISTRATION.

### CONSTRUCTION AND ECONOMICS.

### THE CONTROL OF THE INEBRIATE.

By T. N. KELYNACK, M.D., M.R.C.P., Honorary Secretary of The British Society for the Study of Inebriety.

THE inebriate occupies a foremost place among those decadents of the race for whom control is essential, both for their own protection and the safeguarding of the interests of others. Inebriety may be considered from many standpoints, but no practical study of the condition can be of general service unless the medico-sociological bearings are clearly recognised. Rightly viewed, the alcohol question, as it is commonly termed, is a problem to be investigated in accordance with biological laws and analysed in a strictly scientific spirit. By the application of modern methods of research, directed by the principles of evolution, much hitherto obscure regarding the psychology and physiology of the inebriate has been rendered clear, and with the coming of sound knowledge, ways and means of practical relief have been revealed.

But we are still far from apprehending in anything like completeness the true pathology of inebriety. Its etiology still remains in great part hidden, and we know little as to the best means of securing prophylaxis. The inebriate is no new product of civilisation. The drunkard is a sinner of great antiquity. In every age and among almost all races he has existed, and furnished a source of difficulty and danger to the commonwealth. Numberless systems of treatment have been advocated. "Specifics" for the arrest of inebriety still hustle one another in misleading advertisement. Many clamour for the introduction of "substitutes" for alcohol, bodies which shall arouse the sense of *bien être* without working ill to the human constitution. Numerous bodies vigorously enforce the manifest advantages of the practice of total abstinence, but the majority of people still cling to the use of alcoholic beverages, and regard the widespread lapse from moderation which prevails as no sufficient argument for the relinquishing of intoxicants or their effective restriction.

Much is being done to secure prophylaxis, but comparatively little is being accomplished in the restoration of the inebriate. The recently issued official report of Dr. Welsh Branthwaite, his Majesty's Inspector under the Inebriates Acts, admirably focusses the present situation. This comprehensive Blue Book should be seriously studied by all interested in the pathology of inebriety or having sympathy with efforts directed to procure scientific control of the inebriate.*

The medico-legal aspects of inebriety are crowded with numerous and intricate difficulties. The beginning of modern legislative effort to afford adequate control for the inebriate dates back no further than 1879 when the Habitual Drunkards Act was passed. Since that date much progress, although by slow and short strides, has been made.

At the present time inebriates are being treated in: (1) Certified inebriate reformatories; (2) State inebriate reformatories; and (3) retreats, licensed or unlicensed. In 1899 there were only four reformatories in existence in this country, with 227 available beds, and only 88 committals took place up to the end of the year, all being females. Last year, 1903, there were nine reformatories in full work, with 677 beds and 298 cases were admitted, 39 males and 259 females. At the end of the year 579 cases were under detention, 433 had been discharged on license or were otherwise absent, and the total committals had risen to 1,012.

It should be remembered that the Inebriates Act of 1898 provides powers for the committal of two classes of inebriates for the purpose of control and reformation: (1) Inebriates convicted of crime caused or contributed to by drink. These criminal inebriates may be sentenced direct to a State reformatory or sent to any certified reformatory the managers of which are willing to receive them. (2) Inebriates who have been convicted summarily four times of drunkenness within one year, or of certain other specified offences of which drunkenness forms a part. These police court recidivists can only be committed to a certified reformatory, the Secretary of State having power to transfer such persons from a certified to a State reformatory when this course appears to him to be desirable.

At the present time there are nine reformatories in regular work. Several of these are worked more or less on the Industrial Colony system. An association termed The National Institution for Inebriates carries on useful work at Lewes, Chesterfield, Ackworth, and Horfield. This body arranges with any local authority for the reception of cases committed from courts within its jurisdiction on the execution of a simple agreement to pay maintenance charges and without the necessity for any contribution towards establishment charges or other financial liability. The association seeks to afford the advantages of a series of institutions graded to meet the requirements of different classes of inmates and at the minimum of expense with the maximum of efficiency. Thus the establishment at Lewes serves as a "receiving house" where cases may be thoroughly studied and scientifically sorted; while the home at Horfield provides a "discharging house" for inmates whose sentences will shortly expire. Several new reformatories are nearing completion.

The formation of these institutions is so recent that much in connection with them is necessarily in the experimental stage. Already it is becoming abundantly clear that as with the insane so with inebriates, much discrimination in clinical grouping and strict scientific classification of results are essential if good work is to be accomplished. Religious teaching and moral suasion must be accompanied by strict discipline. The whole atmosphere of the establishment should be reformative. Cases capable of restoration must be separated from the morally irreclaimable and mentally deficient irreformables.

It is very desirable also that the inmates of our inebriate reformatories should be provided with sufficient technical skill so as to be capable of earning a fair livelihood on their discharge. Unless they can be fitted to maintain their place among the ranks of the workers their "cure" is practically worthless from the economic standpoint. Managers of these institutions will do well to face at once this aspect of their work.

Local and judicial authorities are slow to recognise the practical issues raised by the adoption of our modern scientific conception of the nature of inebriety. They tend

* The Report of the Inspector under the Inebriates Acts, 1879 to 1900. For the year 1903. Issued from the Home Office. Pp. 139. London: Eyre and Spottiswoode. 1904.

to cling to punitive measures and forget that the real purpose of legislative control and effective treatment is the reformation of the inebriate and the protection of the State.

Dr. Branthwaite, in his important report, very wisely insists on the importance of adopting a rational system of grouping cases. He advocates the following arrangement:— (1) The removal to State reformatories of the very bad cases, those which prove uncontrollable in certified reformatories; (2) the removal to asylums of the certifiably insane; (3) arrangements, if possible, in a separate reformatory for the proper treatment of the aged, weak-minded, epileptic, and diseased; and (4) a careful selection and isolation from others of the good cases, with a view to their being placed under the best possible conditions most likely to result in the reformation of the largest number.

Classification can be secured (*a*) by the division of a single reformatory into separate buildings; or (*b*) by concerted action between a number of separate institutions, each one being specially designed to take a distinct class of inmate.

There are two State inebriate reformatories, one at Warwick for men and another at Aylesbury for women. In these are maintained the refractory, violent, and weak-minded cases who require management similar to that needed for violent criminals and lunatics.

The constitutional treatment of non-criminal inebriates is far from satisfactory. At the present time there are twenty duly licensed establishments. But, as Dr. Branthwaite points out, "speaking generally, the retreat section of inebriate reform work is not showing that rapid progress which is so evident in connection with the work of reformatories." And with regard to unlicensed homes we would enforce the advice that "It is impossible for intending patients or their friends to discriminate between the good and bad without some guarantee of suitability, such as is provided by licensing and regular inspection. . . . Such persons would be well advised, when selecting an institution, to confine themselves to those which are properly licensed under the Act."

Medical men are often consulted regarding the advisability of trying some of the much-lauded and widely-advertised "cures" for drunkenness. Much harm has undoubtedly been done by the unguarded recommendation of irregular methods of treatment, and both medical men and laymen may well ponder Dr. Branthwaite's evidence on this point. "A reason for the restricted use of retreats is to be found in the multiplicity of patent remedies for drunkenness, promising cure and future immunity after a few days' treatment. Most of these are commercial frauds, which depend for their patronage upon free and alluring advertisements, and upon the advocacy of philanthropic persons who, being non-medical, are incapable of judging the value of facts placed before them, and who are blinded by the plausible representations of the proprietors of such 'cures.' As proof of the extent to which these 'cures' are being resorted to, it is sufficient to say that 37 per cent. of all persons admitted to retreats during 1903 had previously submitted to a secret treatment. The deterrent influence will undoubtedly be temporary, but in the meantime it is, to a certain extent, impeding progress."

Temperance reformers have accomplished much, and many of their rough-and-ready pioneer methods have proved effectual in the restoration of many inebriates. But, if progress is to be maintained and advance secured, it should be clearly realised by all thoughtful minds that the control of the inebriate must be based upon sound scientific principles and guided by well substantiated clinical facts.

We have much yet to learn regarding pathological conditions of the tissues and psychological states of the inebriate. But painstaking experiment and actual experience are demonstrating what is rational and realisable. And it is clear that measures for the control of the inebriate are absolutely essential both for the sake of the individual and the protection of the State.

